# Measurement of Atmospheric CO_2_ Column Concentrations Based on Open-Path TDLAS

**DOI:** 10.3390/s21051722

**Published:** 2021-03-02

**Authors:** Fengxin Xin, Jie Li, Jinjia Guo, Dewang Yang, Yong Wang, Qiuhua Tang, Zhishen Liu

**Affiliations:** 1 Laser Institute, Qilu University of Technology (Shandong Academy of Sciences), Qingdao 266100, China; fengxinxin@sdlaser.cn (F.X.); dewangyang@sdlaser.cn (D.Y.); yongwang@vip.sdlaser.cn (Y.W.); 2 Ocean Remote Sensing Institute, Ocean University of China, Qingdao 266003, China; opticsc@ouc.edu.cn (J.G.); zsliu@ouc.edu.cn (Z.L.); 3 The First Institute of Oceanography, Ministry of Natural Resources of China, Qingdao 266061, China; tangqiuhua@fio.org.cn

**Keywords:** carbon dioxide, column concentration, TDLAS, open-path, comparative measurement

## Abstract

Monitoring of CO_2_ column concentrations is valuable for atmospheric research. A mobile open-path system was developed based on tunable diode laser absorption spectroscopy (TDLAS) to measure atmospheric CO_2_ column concentrations. A laser beam was emitted downward from a distributed feedback diode laser at 2 μm and then reflected by the retroreflector array on the ground. We measured the CO_2_ column concentrations over the 20 and 110 m long vertical path. Several single-point sensors were distributed at different heights to provide comparative measurements for the open-path TDLAS system. The results showed that the minimum detection limit of system was 0.52 ppm. Some similarities were observed in trends from the open-path TDLAS system and these sensors, but the average of these sensors was more consistent with the open-path TDLAS system values than the single-point measurement. These field measurements demonstrate the feasibility of open-path TDLAS for measuring the CO_2_ column concentration and monitoring carbon emission over large areas.

## 1. Introduction

Global warming has become an urgent environmental issue in the world. Anthropogenic emissions of greenhouse gases (GHGs) have a significant impact on global warming, and carbon dioxide (CO_2_) and methane (CH_4_) account for 66% and 16% of radiative forcing, respectively [1,2,3]. During the last decade (2009–2019), CO_2_ concentrations increased at the fastest observed decadal rate of change (2.4 ppm/year), which is higher than that during of any previous decade since direct atmospheric concentration measurements began in 1958 [4,5]. Measurement of the atmospheric CO_2_ column concentrations is valuable for understanding regional carbon emissions, sources, and sinks [6,7,8,9,10,11]. Therefore, it is crucial to measure CO_2_ column concentrations accurately to support ongoing efforts to reduce CO_2_ emissions.

Optical spectroscopy techniques have advantages of high sensitivity, good selectivity, continuous real-time detection, and the noninvasive nature of the measurement [12,13,14,15]. Since the 1990s, optical spectroscopy techniques based on open-path have been employed to measure trace gas column concentrations, such as Fourier transform infrared (FTIR) spectroscopy and differential optical absorption spectroscopy (DOAS). With sunlight, a lamp, or LEDs as the light source, researchers have developed FTIR and DOAS systems for measuring column concentrations of various trace gases simultaneously [16,17,18,19]. However, the broadband light source is disadvantageous to the improvement of spectral resolution, which limits gas detection sensitivity. FTIR and DOAS systems are complex and bulky and thus are not convenient for mobile measurement experiments.

By utilizing low-cost and robust distributed feedback (DFB) laser diodes that offer narrow spectral resolutions, tunable diode laser absorption spectroscopy (TDLAS) has the potential to achieve high sensitivities [20,21,22,23,24]. Open-path TDLAS offers a flexible, cost-effective sensing technology for measuring selected target gases in complex mixtures associated with evolving modern industrial applications [25,26]. Zimmerman et al. (2014) reported the construction and deployment of an open-path TDLAS sensor for pipeline and wellhead monitoring at a carbon capture and storage site [27]. The TDLAS sensor was used to measure CO_2_ concentrations along a fixed 100-m path and proved to be effective for use as an alarm-type system intended for personnel safety. Bailey et al. (2017) have developed an open-path TDLAS instrument with a DFB laser centered near 1572 nm [28]. They reported CO_2_ absorption measurements over a 200-m-long horizontal path to a retroreflector and compared the measurements with that from a single-point sensor at the field site; similar trends in diurnal cycles of CO_2_ concentration were observed. Xia et al. (2019) presented measurements of CO_2_ and CH_4_ over a 2.6-km-long horizontal path using an open-path TDLAS instrument [29]. The sensitivities of the instrument for CO_2_ and CH_4_ were evaluated as 20 ppm and 20 ppb, respectively. In addition, several other researchers have measured the average concentration of gases over a horizontal path. However, until now, open-path TDLAS was rarely used to measure the column concentration of trace gases along the vertical direction. Considering the advantages of open-path TDLAS, it is of great significance to apply it to the measurement of atmospheric CO_2_ column concentration for the long-term monitoring of CO_2_ over a large area. In the future, it can be used for all-day monitoring of CO_2_ column concentration through the appropriate meteorological observation stations and to analyze the spatial and temporal variation of CO_2_ in the regional atmosphere. Furthermore, it also helps us to control the CO_2_ emissions within a region, and even understand the climate change of the region. Therefore, measurement of CO_2_ column concentration in a large number of regions based on open-path TDLAS is valuable for improving climate warming.

In this paper, we present measurements of atmospheric CO_2_ column concentrations with an open-path TDLAS system that operates on a 2002.51 nm line. Direct absorption was further verified and was simpler and lower-cost than wavelength modulation spectroscopy, while achieving essentially calibration-free measurements. Hence, it was applied to establish the open-path TDLAS system. Atmospheric CO_2_ column concentrations over the 21 and 110 m long vertical path were measured. The signal-to-noise ratio (SNR) and minimum detection limit of the system were calculated to be 880 and 0.52 ppm. During the monitoring period, several single-point sensors were used to estimate the average concentration over the vertical path and compared with the open-path TDLAS system.

## 2. Theoretical Principle

**Figure 1:**
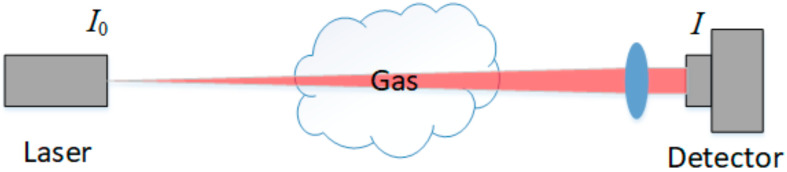
Laser absorption spectroscopy concept.

When a laser beam passes through the gas medium, the intensity (*I*_0_) of the incident laser will decay exponentially due to the absorption of gas (Figure 1). The intensity (*I*) of the received laser on the detector can be described by the Lambert–Beer law.

(1) $$I(v)=I_{0}e^{-k(v)L}$$

where *k*(*v*) [cm^−1^] is the spectral absorption coefficient, expressed as

(2) k(v)=PS(T)f(v)C

where *P* [atm] is the gas pressure; *S*(*T*) [cm^−2^atm^−1^] is the absorption line strength; *f*(*v*) [cm] is the line shape function, which can be normalized after modulated by a low frequency ramp voltage, i.e., $\int_{-\infty }^{+\infty }f(v)dv=1$; *C* [ppm] is the gas concentration. Therefore, we can obtain the integral absorbance, *τ* [cm^−1^], which can be expressed as

(3) $$\tau =\int_{-\infty }^{+\infty }\ln\left(\frac{I_{0}}{I}\right)dv=PS(T)LC$$

We can get *S* (*T*) [cm/molecule] in the HITRAN database, and it can be converted to *S* (*T*) [cm^−2^atm^−1^] as shown in Equation (4). By calculating the integrated absorbance (*τ*), the absorption line intensity (*S* (*T*)), the gas pressure (*P*), and the optical path (*L*), we can obtain the concentration of CO_2_ as follows.

(4) $$S\left(T\right)\left[{\mathrm{cm}}^{-2}\cdot {\mathrm{atm}}^{-1}\right]=S\left(T\right)\left[\mathrm{cm}/\mathrm{molecule}\right]\cdot \frac{7.34\times 10^{21}\left[\mathrm{molecule}\cdot K\right]}{T\left(K\right)\left[{\mathrm{cm}}^{3}\cdot \mathrm{atm}\right]}$$

(5) $$C=\frac{\tau }{PS(T)L}$$

The direct absorption signal measurement method is simple and well verified, and it does not require to be calibrated with standard gas at different concentrations. Therefore, it can readily be applied in an open-path TDLAS experimental system.

## 3. Experimental Configuration

### 3.1. Selection of Carbon Dioxide Absorption Bands

**Figure 2:**
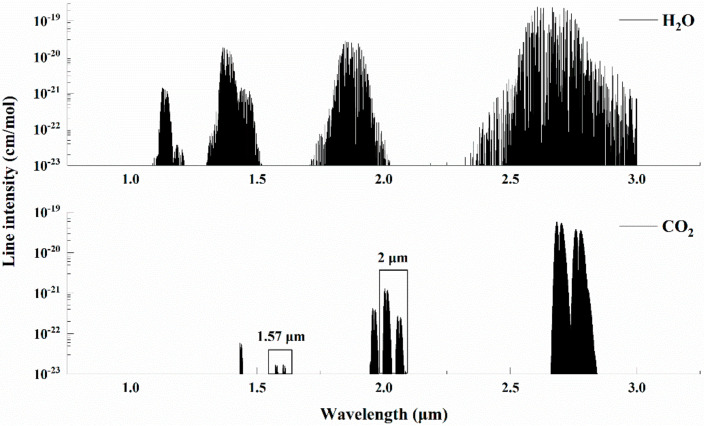
The absorption spectrum of CO_2_ and H_2_O between 1.0 μm and 3.0 μm.

**Figure 3:**
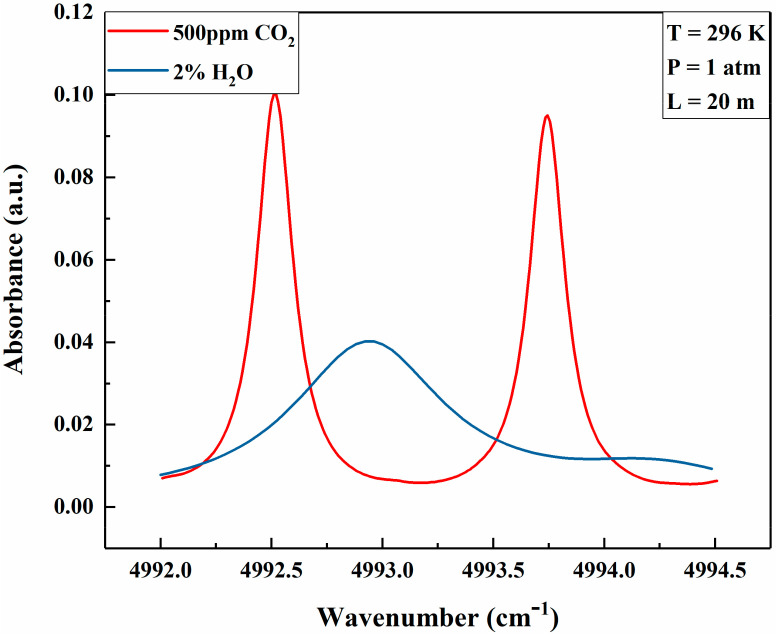
Simulated absorbance of CO_2_ and H_2_O with the HITRAN database.

The absorption band of CO_2_ was selected by considering the absorption line intensity and minimal interference from other species in the ambient air. Figure 2 shows the absorption bands of CO_2_ and H_2_O in the IR region based on the HITRAN2012 database [30]. As can be seen from Figure 2, CO_2_ absorption bands near 1.57 μm and 2 μm are free of H_2_O interference and can be used for measurements of CO_2_. However, the absorption line intensity of CO_2_ near 2 μm is at the level of 10^−21^ cm/mol, which is 2 orders larger than that of 1.57 μm, and thus is suitable for the direct absorption spectroscopy. The simulated absorbance of H_2_O and CO_2_ for a temperature of 296 K, 1 atm pressure, and 20 m optical path-length with fractional volumes of 2% of H_2_O and 500 ppm of CO_2_ (typical for ambient air) in the range of 4992 cm^−1^ to 4994.5 cm^−1^ were calculated with the HITRAN database, as shown in Figure 3. It was found that the absorbance located at 4992.52 cm^−1^ (2003.00 nm) and 4993.74 cm^−1^ (2002.51 nm) were close to each other, but the absorption line located at 4993.74 cm^−1^ had less overlap with H_2_O than that of 4992.52 cm^−1^. Therefore, the absorption line of CO_2_ centered at 4993.74 cm^−1^ (2002.51 nm) was selected in this research.

### 3.2. Experimental Set-Up

**Figure 4:**
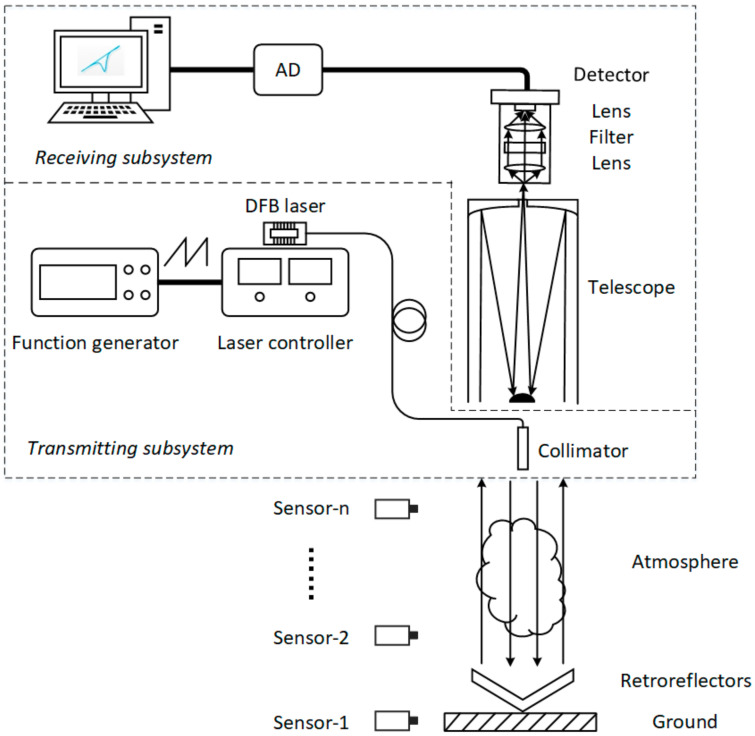
Schematic diagram of the open-path tunable diode laser absorption spectroscopy (TDLAS) system for measuring atmospheric CO_2_ column concentration.

The schematic diagram of the open-path TDLAS system is shown in Figure 4. A single-mode continuous DFB laser (Nanosystems and Technologies GmbH, Gerbrunn, Germany) with a fiber tail was selected as the laser source, with a wavelength of ≈2 μm corresponding to the absorption spectrum of carbon dioxide; the output power was 5.8 mW. A commercial laser diode controller (LDC-3724C, ILX Lightwave, Monroeville, AL USA) was selected as the laser driver to control the temperature and current of laser with high stability and low noise. After it was tuned by a ramp signal from a function generator, the laser beam was emitted downward to the retroreflector array on the ground, which was composed of seven same-size retroreflectors of 64 mm in diameter. The reflected laser beam was received by a telescope before passing through a lens and a bandpass filter with a 10 nm spectral width and focused by a lens on a PIN photodiode detector (PDA10D-EC, Thorlabs Corp., Newton, NJ, USA). The detector converted the received optical signal into an electrical signal. The electrical signal was acquired by an AD acquisition card (NI 9215, National Instruments, Roscoe, IL, USA) at 16-bit, and CO_2_ concentration was obtained after processing the data. To assess the accuracy of CO_2_ concentrations in a vertical open-path measured by the TDLAS system and the differences of atmospheric CO_2_ concentrations at different heights, several single-point sensors were placed at different locations along the vertical path.

## 4. Measurements and Results

**Figure 5:**
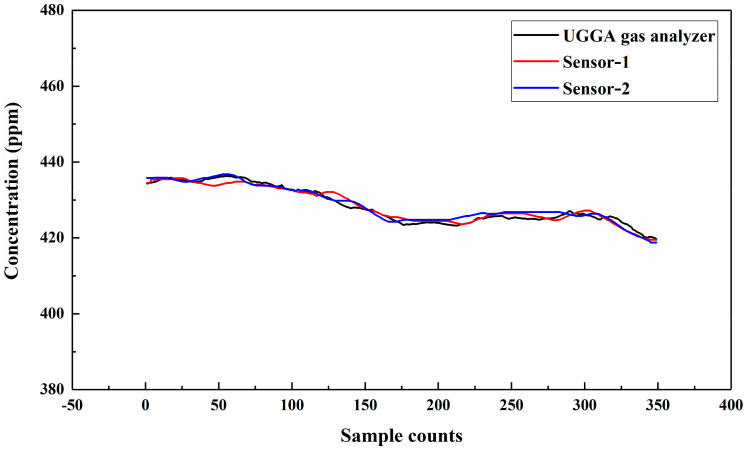
AZ7752 Sensors calibration results with UGGA gas analyzer.

The open-path TDLAS system transmitted a laser vertically toward the ground through the window of a laboratory located at Ocean University of China, Yushan campus (36.0685° N, 120.3435° E). The path length measured with a laser rangefinder was 21 m. Two handheld non-dispersive infrared single-point sensors (AZ-7752, AZ Instrument Corp., Taipei, Taiwan) were used to measure CO_2_ concentrations at different heights. One was placed on the ground and the other was placed around the system, which were called sensor-1 and sensor-2, respectively. To ensure the accuracy of measurement results, a commercial gas analyzer (UGGA, Los Gatos Research, San Hose, CA, USA) was used to calibrate each AZ7752 sensor based on off-axis integration cavity output spectroscopy (OA-ICOS). Results of the calibration experiment can be found in the author’s previous study [31]. The numerical deviation between two sensors was within 3 ppm, as shown in Figure 5.

**Figure 6:**
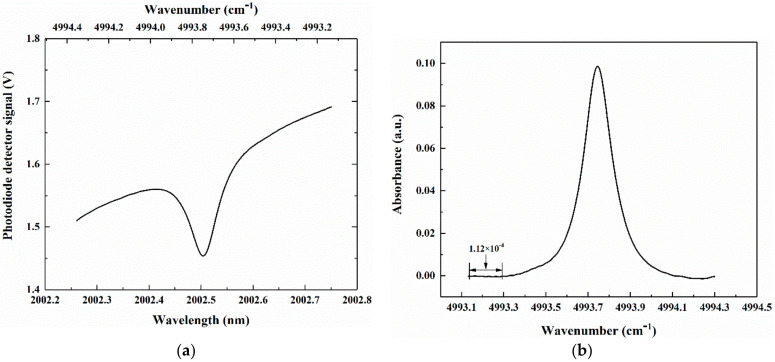
Received signal from photodiode detector (**a**) and measured absorbance of CO_2_ (**b**).

The temperature and current of the laser were controlled to be 31.4 °C and 90 mA, respectively. The ramp voltage amplitude varied from −350 mV to 500 mV, so that the absorption line of CO_2_ that was centered at 4993.74 cm^−1^ occurred in the range of the scan, as shown in the signal received using the photodiode detector (Figure 6). The 1-σ background noise was determined to be 1.12 × 10^−4^, by calculating the standard deviation of the non-absorption wing of measured CO_2_ absorbance. The SNR of 880 was determined based on the ratio of the maximal amplitude of measured CO_2_ absorbance to the 1-σ background noise. In addition, the integral absorbance (*τ*) was obtained to be 2.5 × 10^−2^, and CO_2_ concentration was calculated to be 458.89 ppm, according to Equation (5). Therefore, the minimum detection limit was 0.52 ppm, which confirms that the system can be used to measure the atmospheric CO_2_ concentration.

**Figure 7:**
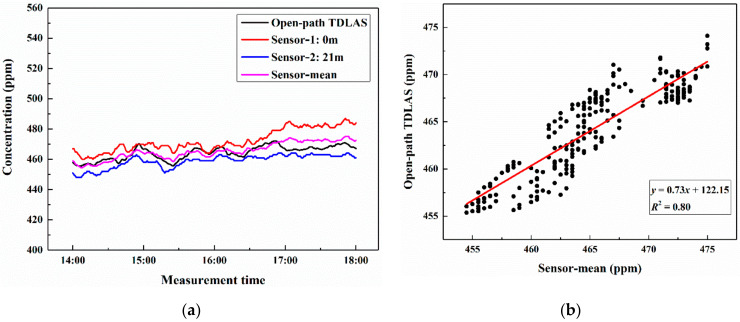
Atmospheric CO_2_ concentrations over the 21-m-long vertical path (**a**) and comparison of open-path TDLAS with the average values of two sensors (**b**).

The measured CO_2_ concentrations over the 21-m-long vertical path were presented in Figure 7a. During the measurement time, the outdoor weather was fine and there was no rain or snow and other bad weather. The temperature was in the range of 31.1 °C to 30.2 °C, and the atmospheric pressure was stable at 100.1 kPa. The results showed that the measured concentration of the open-path TDLAS system and the two sensors have a consistent variation trend, but there were some differences in the values. Under the influence of atmospheric diffusion, CO_2_ molecules were not uniformly distributed in the vertical direction. As the height increases, CO_2_ concentration was less affected by ground emission sources. Therefore, the concentration measured by sensor-2 located at 21 m height was lower than that measured by sensor-1 on the ground. The results of the TDLAS system were larger than the average atmospheric value (≈410 ppm) because CO_2_ concentration is susceptible to human activities and vegetation on campus within the 21-m-long vertical measurement path. These values varied between 455 ppm and 475 ppm, which were similar to the average values (sensor-mean) of the two sensors’ measurements. Correlation analysis was conducted between the TDLAS system and sensor-mean, and the determination coefficient (*R*^2^) was 0.8, as shown in Figure 7b, which indicates that the concentration measured by them has good consistency.

**Figure 8:**
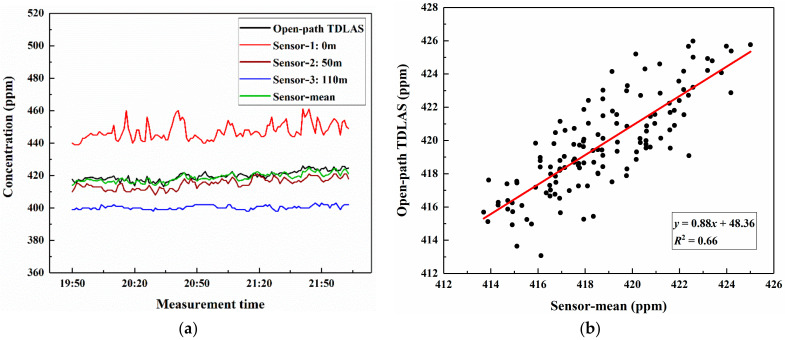
Atmospheric CO_2_ concentrations over the 110-m-long vertical (**a**) and comparison of open-path TDLAS with the average values of three sensors (**b**).

To estimate the atmospheric CO_2_ column concentration over a large area, we carried out an experiment over a 110-m-long vertical path. The open-path TDLAS system was moved to a residential building located in the Binhai Garden community (36.0693° N, 120.3888° E). The collimated laser was transmitted vertically downward through a window on the 33rd floor. In addition to the sensors on the ground and at the top of the vertical path, an AZ7752 sensor was placed on the 15th floor to measure the atmospheric CO_2_ concentration at a 50 m height. The weather was fine, with temperature and pressure stable at 24.2 °C and 100.8 kPa, respectively. As shown in Figure 8a, the CO_2_ column concentration measured by the TDLAS system was about 420 ppm and remained almost constant, showing smaller relative variations compared to that over the 21-m-long vertical path. The results indicated that the values of the TDLAS system were markedly different from that of the three sensors. Due to interference by passing cars and pedestrians, some sharp spikes occurred in the results of sensor-1. However, as shown by sensor-2 and sensor-3, CO_2_ concentration and its fluctuation amplitude decreased with the increase of height, so the results of each sensor were not representative of the average concentration over the large area. Furthermore, the column concentrations measured by the TDLAS system were close to the average values of the three sensors in this experiment, and the determination coefficient (*R*^2^) was also calculated with a value of 0.66, as depicted in Figure 8b.

## 5. Discussion

Through the above two groups of CO_2_ column concentration measurements with vertical paths of different length, the continuous monitoring of CO_2_ concentration over different scales were carried out for a few hours. The results of previous open-path techniques research and our work are summarized in Table 1. The minimum detection limit of the system was calculated to be 0.52 ppm, which is better than previous research, indicating that the feasibility of detecting CO_2_ concentration along the vertical direction based on open-path TDLAS. In addition, we will increase detection distance to measure CO_2_ concentration over larger areas.

The average of multiple single-point sensors located at different heights was applied to denote CO_2_ concentrations in the region approximately. Compared with the results of each sensor, the average of these sensors was more consistent with the column concentration measured by the TDLAS system. The determination coefficient between TDLAS and sensor-mean over the 110-m-long vertical path was not as good as that over the 21-m-long vertical path. Partially, the three sensors may not adequately represent the average CO_2_ concentration over the large area. In the future, we will use more point sensors and do more work on monitoring CO_2_ concentration over large areas based on open-path TDLAS.

## 6. Conclusions

In this paper, an open-path measurement system was built to measure atmospheric CO_2_ column concentrations based on TDLAS. The minimum detection limit was evaluated to be 0.52 ppm, indicating the feasibility of open-path TDLAS system. The atmospheric CO_2_ column concentrations over a 21-m-long vertical path and a 110-m-long vertical path were monitored within a few hours, respectively. The results showed that the column concentration over the 110-m-long path was more stable than that over the 20-m-long path, meeting the average value of atmospheric CO_2_. Several single-point sensors were placed in different positions of the vertical path to carry out the comparative experiment. The difference of these sensors’ readings indicates the non-uniformity of CO_2_ concentrations in the vertical direction. The average of several sensors was consistent with the result of the TDLAS system, and the determination coefficients were 0.8 and 0.66 in the two sets of experiments. Their correlation can be improved by increasing the number of sensors for future experiment over large areas. The developed open-path TDLAS system provides a convenient way of measuring the CO_2_ column concentration in the regional atmosphere and the possibility for monitoring CO_2_ emission over a large area.

## Figures and Tables

**Table 1:** Comparisons with previous open-path spectroscopy techniques for detection CO_2_.

Techniques	Light Source	Distance	Direction	Detection Limit	Comparison Sensors	Reference
FTIR	Lamp	1.5 km	Horizontal	1.6 ppm	Single	[16]
DOAS	LED	3.045 km	Horizontal	-	-	[19]
TDLAS	DFB laser	0.2 km	Horizontal	5.4 ppm	Single	[28]
TDLAS	DFB laser	1.3 km	Horizontal	20 ppm	-	[29]
TDLAS	DFB laser	0.11 km	Vertical	0.52 ppm	Multiple	This work

## Data Availability

Data sharing not applicable.
